# Larvicidal Effect of *Vorticella microstoma* (Ehrenberg, 1830) on Mosquito Larvae, and Morphological Changes under Induced Environmental Conditions

**DOI:** 10.1155/2020/5659808

**Published:** 2020-09-01

**Authors:** Achini Koshila Ranasinghe, L. D. Amarasinghe

**Affiliations:** Department of Zoology and Environmental Management, Faculty of Science, University of Kelaniya, Dalugama, Kelaniya 11600, Sri Lanka

## Abstract

Development of microbiota assemblage usually occurs in all most all domestic and peridomestic mosquito breeding habitats. There may be parasitic, epibiont, pathogenic, or even predatory species among this biota, and to investigate their potential against the mosquito population is worth studying. This may contribute to formulating environmentally agreeable approaches in controlling mosquitoes which is a current need. *Vorticella* spp. is a peritrich ciliate, and its trophont stage has become epibiont to certain biota. Further, their existence in seasonal water bodies that dry off during drought in tropical weather conditions is not known. Therefore, the potential of the larvicidal effect of *Vorticella microstoma* on different species of mosquito larvae was studied. We found that *V. microstoma* causes the 100% death of the third instar larvae of *Culex tritaeniorhynchus* (Giles, 1901) within 48 h of exposure. In contrast to that, this species did not cause any mortality to *Aedes albopictus* (Skuse, 1894) and *Aedes aegypti* (Linnaeus in Hasselquist, 1762) mosquito larvae in repeated trials. The dynamics of polymorphism of *V. microstoma* was studied under induced environmental conditions. *V. microstoma* remained as trophont stage throughout at room temperature (25 ± 2°C). When the temperature was reduced to 6°C, *V. microstoma* settled in the cyst stage. Evidently, *V. microstoma* is a good biocontrol agent of *Culex* species mosquito larvae, and they able to overcome drought periods in cyst forms. The findings of this study would be considered as the first step for a new avenue to work on environmentally agreeable manner in reducing the *Culex* spp. mosquito populations.

## 1. Introduction

Mosquitoes transmit most of the life-threatening diseases like malaria, filariasis, Japanese encephalitis, dengue fever, chikungunya fever, yellow fever, West Nile virus infection, and ZIKA fever [1]. Therefore, mosquito control is essential to prevent the proliferation of mosquito-borne diseases and to improve the quality of the environment and public health. The best approach is either killing adult mosquitoes preventing them from biting people or by killing the larvae at breeding sites, to interrupt the disease transmission [2].

The common mosquito larvicides are organophosphates and pyrethroids. However, the effectiveness of the vector control by the synthetic insecticides has declined due to the development of resistance in mosquitoes to currently used insecticides [2]. In addition to that, the health risks for human and domestic animals and disturbances to the natural balance such as predator-prey or parasite-host relationships warrant formulating environmentally agreeable approaches in controlling mosquitoes.

There is always a varying level of microbiota assemblage among the biotic factors in all most all these mosquito breeding habitats. Among these living beings, there may be free-living, parasitic/epibiont, pathogenic, or even predatory species that can affect the life of the developing mosquito immatures. Some of the biota serve as food items of the larvae, while some may serve as parasitic/epibiont in living in the body of the mosquito larvae, and some may serve as predators [3].

*Vorticella* Ehrenberg is a suspension-feeding ciliate that lives in two forms: the free-swimming telotroch and the sessile stalked trophont [4]. The stalked *Vorticella* has contractile myonemes, allowing them to pull the cell body against substrates [5]. A sessile *V. microstoma* consists of a single zooid, body vase-like, with a long contractile stalk [6].

Further, *Vorticella* sp. is a microbiota species associated with mosquito breeding habitats in cultivated and abandoned rice fields of Sri Lanka [3], and it has been found to occasionally infect mosquito larvae in other countries [7–10]. *Vorticella* sp. has been explored as a biocontrol agent of mosquitoes recently [11].

Warren [12] has reported the formation of a cyst around the body of *Vorticella* during unfavorable conditions. Further, Warren [12] reports that the encysted body breaks off from the stalk. In this condition, *Vorticella* tides over the unfavorable conditions. After the return of favorable conditions, the cyst breaks and the individual emerges, develops a contractile vacuole, and becomes enlarged. It grows an aboral circlet of cilia to become a telotroch. It swims freely for some time and then settles on some substratum, develops a stalk, and grows into an adult *Vorticella.*

Therefore, the present study was conducted to (1) maintain and cultivate *V. microstoma* collected from natural mosquito breeding habitats under laboratory conditions; (2) determine the larvicidal effect of *V. microstoma* to selected mosquito species and instar levels; (3) determine the different morphological forms of *V. microstoma* under induced environmental conditions.

## 2. Materials and Methods

### 2.1. Field Collection of *Vorticella microstoma* and Maintenance in the Laboratory

*V. microstoma* used in this study were originally recovered together with moribund and dead *Culex tritaeniorhynchus* larvae in 200 mL water samples collected from a cultivated paddy field in Melsiripura in Kurunegala district, Sri Lanka (GPS location: 7°37.579′N, 80°29.618′E). This site was identified as *V. microstoma* positive selected abiotic variables, namely; pH and dissolved oxygen (DO) of water of the sampling site were measured using a multiparameter (HACH-HQ40d) in a manner that ¼ of the probe is dipped in water, *in situ*. Determination of the five-day biological oxygen demand (BOD5) was carried out as described in APHA [13]. pH, DO, and BOD of the sampling site were measured as 7.15, 6.99 mg L^−1^_,_ and 6.87 mg L^−1^, respectively.

Water samples from the paddy field (*n* = 15) were brought into the laboratory in plastic containers, with *Cx. tritaeniorhynchus* mosquito larvae. After two days, the dead *Cx. tritaeniorhynchus* larvae were collected and observed under a microscope (OLYMPUS x C21; Jeff Liu Ningbo Huasheng Precision Technology International Trading Co., Zhejiang, China). The dead larvae having *V. microstoma* attached to their bodies were transferred at the rate of five larvae into wide-mouthed plastic bottles (height: 12 cm, width: 6.5 cm) filled with 50 mL distilled water. Ten well-cleaned and dried, 2.5 cm-long pieces of hay were placed in each bottle as a substratum for the attachment of *V. microstoma*, while they are multiplying. The mouth of the bottles were individually covered with a small-sized mesh net and maintained for four days at room temperature (25 ± 2°C) under laboratory conditions. The mean number of *V. microstoma* trophonts attached to a single piece of hay in a culture bottle was 60 ± 5 at the initial stage. However, the mean number of trophonts contained in one culture bottle after 4 days period was estimated as 2500 ± 300. A series of *V. microstoma* culture bottles were prepared every five days to prevent the growth of other microbiota species, such as *Philodina citrina* (Rotifera) (Ehrenberg, 1832) and *Paramecium* (Müller, 1773). One such *V. microstoma* culture bottle was considered as an experimental unit and used for experimentations, as described in this study.

### 2.2. Collection of Mosquito Larvae and Species Identification

*Aedes albopictus*, *Aedes aegypti*, *Cx. tritaeniorhynchus*, *Culex gelidus* (Theobald, 1901), and *Tripteroides* spp. (Giles, 1904) larvae were collected from Kelaniya (06°58.426′N, 79°54.939′E), Ragama (07°02.660′N, 79°55.957′E), Kurunegala (7°35.510′N, 80°26.413′E), Nahena (6°59.707′N, 79°54.758′E), and Alawwa (7°18.493′N, 80°15.712′E), respectively. Species identification was confirmed by standard identification guides of mosquito larvae [14–16].

### 2.3. Larvicidal Rate of *V. microstoma* on Mosquito Larvae

The larvicidal assay was performed according to the guidelines of WHO [17]. Fifteen third-instar mosquito larvae were introduced into *V. microstoma* culture bottles (*n* = 10) at room temperature. After 48 h of exposure, the number of dead mosquito larvae was counted. Dead larvae were picked using a pasture pipette and placed on a microscopic glass slide containing a drop of saline. Thereafter, they were observed under a microscope (OLYMPUS x C21; magnification 100x). Larvae infected with *V. microstoma* were identified by the presence of epibionts attached to the body surface. *Culex tritaeniorhynchus*, *Ae. albopictus*, and *Tripteroides* spp. larvae, and a combination of equal numbers of two mosquito species, *Cx. tritaeniorhynchus* and *Tripteroides* spp., were tested. One set of control was maintained for each treatment.

Fifteen each of the first, second, and third instar larvae of *Cx. gelidus* and *Ae. aegypti* were placed in separate *V. microstoma* culture bottles at room temperature. Three replicates were run for each instar level. After 24 and 48 h, the number of dead mosquito larvae was counted. The dead larvae were observed under a microscope (OLYMPUS x C21; magnification 40x), and heavily infested larvae with *V. microstoma* were identified by the presence of epibionts attached all over the body surface. One set of controls were maintained for each treatment.

### 2.4. Effects of Variation in Temperature and Dehydration on the Different Morphological Forms of *V. microstoma*

*V. microstoma* trophont stage colony formed on 2.5 cm-long hay pieces from 4 days old culture bottles were collected and one piece was placed in each petri dish of diameter 9 cm and depth 0.8 cm (*n* = 18). They were placed under six different conditions in three replicates.

1. At room temperature (25 ± 2°C), 30 mL of distilled water was added into the dish
2. At room temperature (25 ± 2°C), no water was added; dehydration was induced
3. At 11°C in a bottle cooler, 30 mL of distilled water was added into the dish
4. At 11°C in a bottle cooler, no water was added; dehydration was induced
5. At 6°C in a refrigerator, 30 mL of distilled water was added into the dish
6. At 6°C in a refrigerator, no water was added; dehydration was induced

Changes in the morphology of *V. microstoma* were observed under the microscope after 24 h.

### 2.5. Viability of the Cyst Stage of *V. microstoma* under Prolonged Dry Condition

The cysts of *V. microstoma* formed on hay pieces under dehydrating conditions in the previous experiment were placed individually on a filter paper placed at the bottom of petri dishes with diameter 9 cm (*n* = 10), thus allowing the cysts to continuously dry at room temperature (25 ± 2°C). In each 24 h interval, one petri dish was taken and 5 ml of water was added, and this was continued for 21 days. Morphological changes to *V. microstoma* cyst after water addition were observed after 24 h, and the data were recorded.

### 2.6. Data Analysis

Mosquito larval mortality data were analyzed using IBM SPSS Statistics 22 software (developed by International Business Machines Corporation-IBM, US). The mortality effect of *V. microstoma* on different mosquito species and between different larval instar levels were analyzed using one-way ANOVA and post hoc comparisons.

## 3. Results

### 3.1. Mosquito Larvicidal Effect of *V. microstoma*

**Figure 1:**
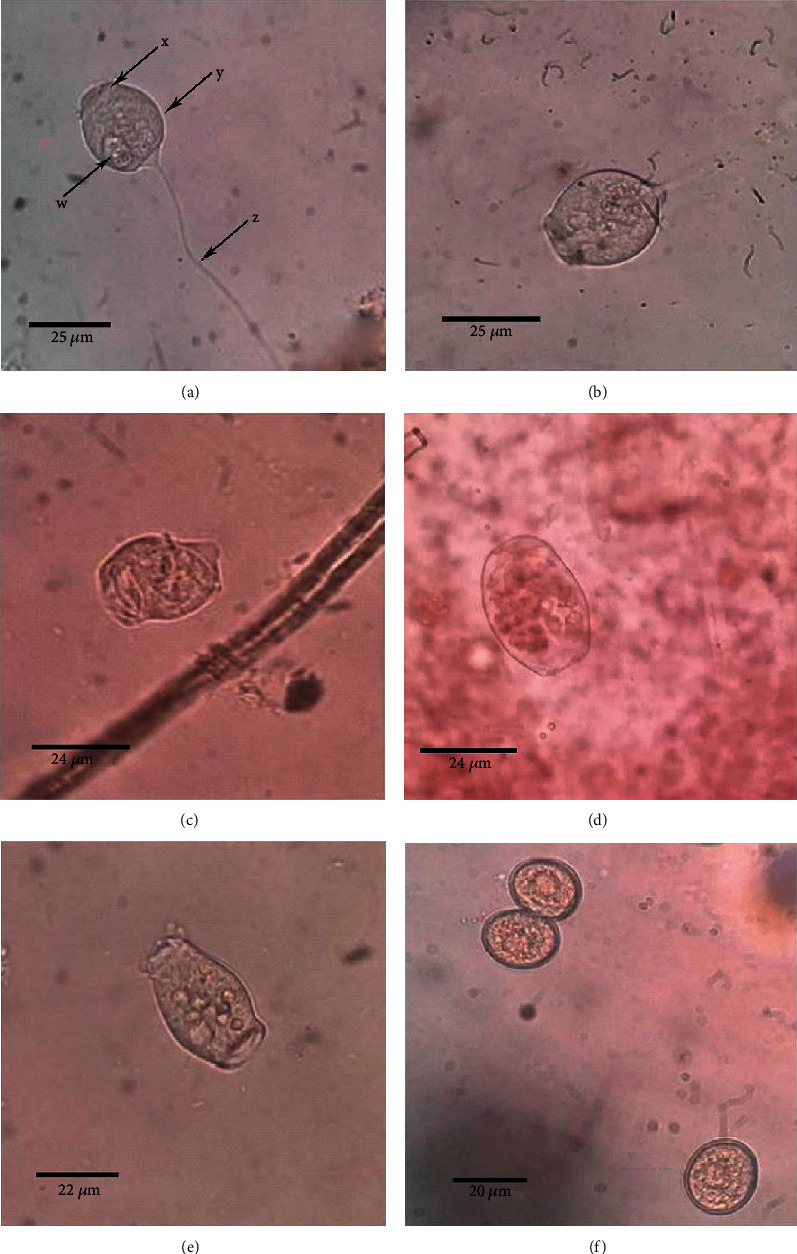
Different morphological forms of *V. microstoma* ((a) sessile stalked trophont stage (w: contractile vacuole, x: oral cilia, y: cup-like body, c: contractile stalk), (b) detached cup-like body with a short stalk, (c) detached cup-like body without a stalk, (d) intermediate stage between cup-shape and elongated shape, (e) elongated telotroch stage, (f) cyst stage) (×400).

**Figure 2:**
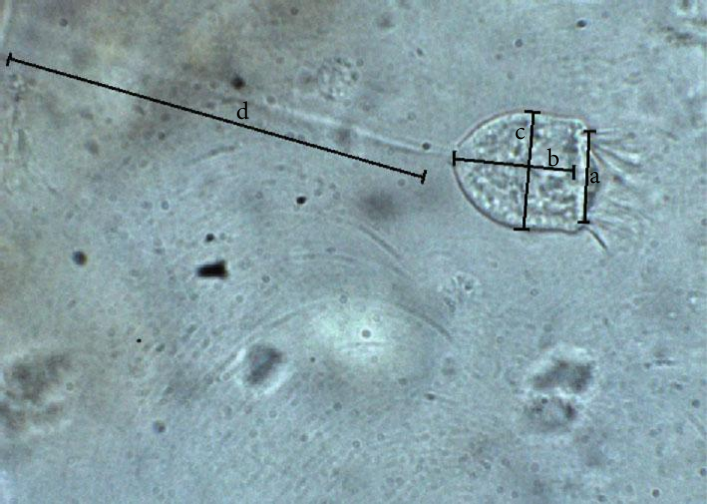
Microscopic view of the trophont stage of *V. microstoma* ×400 ((a) width of the open peristome; (b) length of the body; (c) width of the body; (d) length of the contractile stalk).

The mosquito larvae were observed under a microscope (40x magnification) and identified as infested with an epibiont, which is the live sessile stalked trophont stage of *V. microstoma*, with a cup-shaped body and a contractile stalk attached to the substrate (Figure 1(a)). Speciation was performed using key morphological characteristics, the body is vase-like; slightly yellowish; anterior region (= “peristome”) with buccal ciliation that winds counterclockwise to the buccal cavity; anterior region rather narrow (Figure 1(a)) by comparison with other species of the genus; one long band form macronucleus extending more or less along the longitudinal axis of the cell; a single micronucleus; a contractile vacuole is located in the buccal cavity; usually solitary, although sometimes in large groups. Mature sessile individuals without body ciliation were found [18]. Body length: 49.984 *μ*m ± 3.41, body width: 27.098 *μ*m ± 1.42, the width of open peristome: 19.74 *μ*m ± 3.10, and the length of the contractile stalk: 80.23 *μ*m ± 14.94 (Figure 2) were observed.

**Figure 3:**
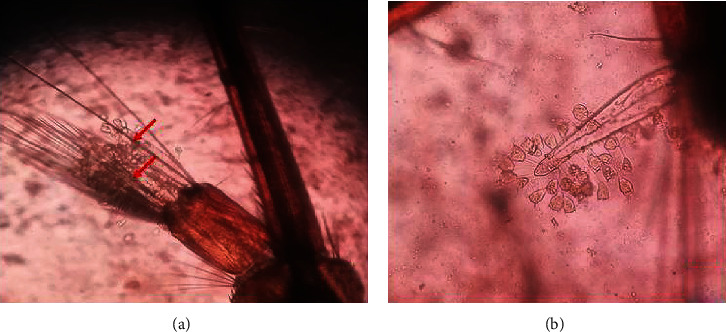
Infection of the parasite (*V. microstoma*) to 3^rd^ larval instars of *Cx. tritaeniorhynchus* anal papillae region (×40 magnification), and attached trophonts of *V. microstoma* (×100 magnification).

**Figure 4:**
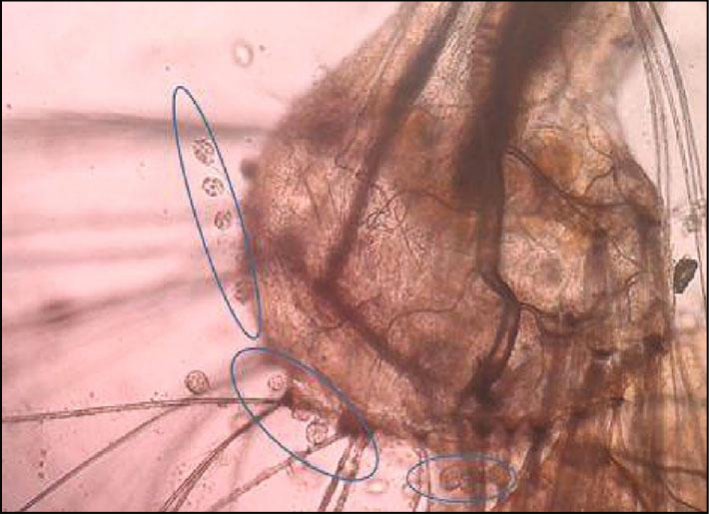
*V. microstoma* infected dead *Cx. tritaeniorhynchus* 3^rd^ instar larvae head region (×400) (attached trophonts to the body are shown inside circles of blue color).

Higher densities of this organism were attached to the saddle and head regions (Figures 3 and 4), followed by the abdominal regions of the body of dead mosquito larvae. *V. microstoma* usually did not attach to the siphon region of live mosquito larvae; instead, they attached to other regions of the body. However, *V. microstoma* attached to the siphon and head regions once the larvae died.

**Figure 5:**
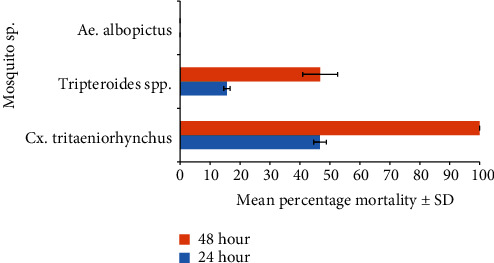
Mean percentage mortality of mosquito larvae 3^rd^ instars due to *V. microstoma* attachment. Control values remained zero mortality.

*Culex tritaeniorhynchus* was the most preferred host of the trophont stage of *V. microstoma*, causing the death of 100% of the mosquito larvae, followed by *Tripteroides* spp., in which 46.7% of the larvae died. *Aedes albopictus* larvae were not preferred by *V. microstoma*. Therefore, the mortality rate of *Ae. albopictus* due to *V. microstoma* was zero (Figure 5). The mean mortality percentage of the mosquito species studied were significantly different from each other (one-way ANOVA: 0.001 < *P* < 0.05, *F* = 24.143). According to multiple comparisons, the mortality percentage of *Cx. tritaeniorhynchus* was significantly higher than that of *Tripteroides* spp. and *Ae. albopictus* larvae (one-way ANOVA post hoc comparisons Sig. 0.010 < 0.05, 0.000 < 0.05, respectively) (Table 1).

*Culex tritaeniorhynchus* larvae showed more susceptibility than *Tripteroides* spp. larvae did for infection by *V. microstoma* when both these species were kept together; however, the mortality did not differ significantly from each other (one-way ANOVA: *P* = 0.374 < 0.05, *F* = 1.000) showing that there is a reducing tendency in the of mortality of *Cx. tritaeniorhynchus* (33.3 ± 11.54) compared to that of *Tripteroides* spp. (20 ± 10).

### 3.2. Susceptibility of *Cx. gelidus* and *Ae.aegypti* Larval Instar Levels to *V. microstoma*

**Figure 6:**
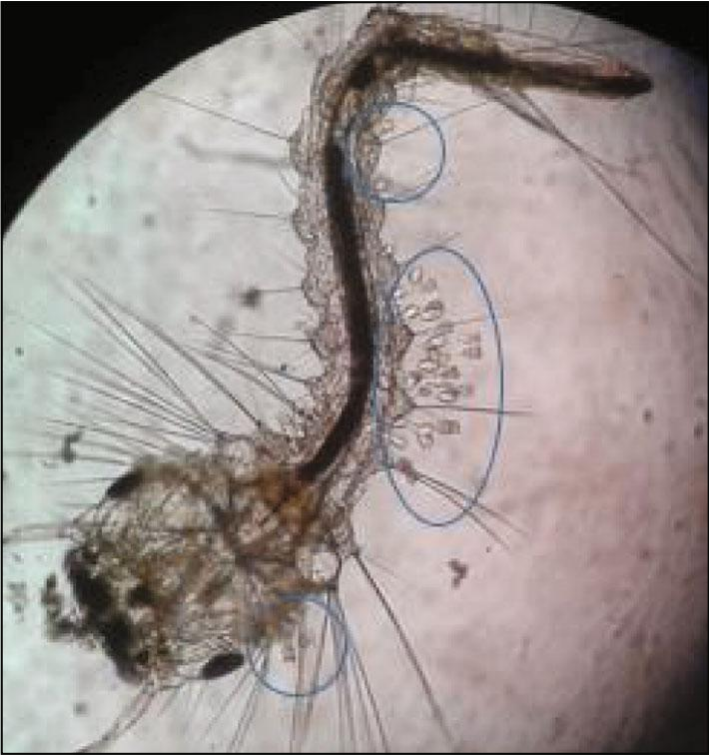
*V. microstoma* infected thoracic and abdominal region of 1^st^ instar larvae of *Cx. gelidus* ×40) (attached trophonts to the body are shown inside circles of blue color).

**Figure 7:**
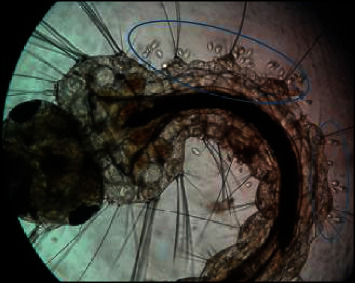
*V. microstoma* infected abdominal region of 2^nd^ instar larvae of *Cx. gelidus* (×40) (attached trophonts to the body are shown inside circles of blue color).

**Figure 8:**
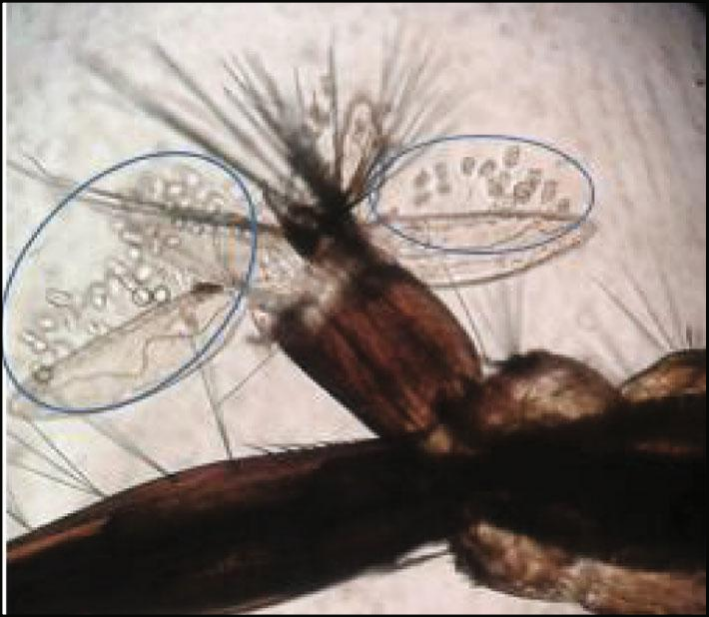
*V. microstoma* infected anal papillae of 3^rd^ instar larvae of *Cx. gelidus* (×40) (attached trophonts to the body are shown inside circles of blue color).

**Figure 9:**
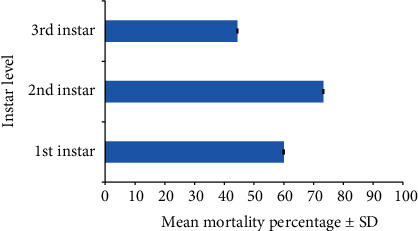
Mean mortality percentage ± SD of different instar levels of *Cx. gelidus* larvae.

The first, second, and third instar larvae of *Cx. gelidus* were infested with the trophont stage of *V. microstoma.* None of the instar levels of *Ae. aegypti* showed susceptibility to infection with *V. microstoma*. In the first instar larvae of *Cx. gelidus*, 30–35 *V. microstoma* got attached with higher densities found in the thoracic region followed by the other segments (Figure 6), whereas 2–4 trophonts were attached to the anal papillae. In the second instar larvae of *Cx. gelidus*, 45–60 *V. microstoma* got attached, with higher densities in abdominal segments (Figure 7); in anal papillae, 5–6 trophonts were attached. In the third-instar larvae of *Cx. gelidus* 50–85 *V. microstoma* got attached, with higher densities in the anal papillae followed by abdominal segments (Figure 8); in the anal papillae, 20–25 trophonts were found to be attached. Mean mortality percentage of the different instars of *Cx. gelidus* larvae are shown in Figure 9.

The percentage mortality of the first, second, and third instar larvae of *Cx. gelidus* did not significantly differ from each other (one-way ANOVA: *P* = 0.298 < 0.05, *F* = 1.494). Multiple comparisons (one-way ANOVA post hoc comparisons) of mortality percentage of the first, second, and third-instar of *Cx. gelidus* larvae did not reveal significant differences as compared with that of all the other instar levels (Table 2). The mortality values of the controls remained zero.

### 3.3. Effects of Variation in Temperature and Dehydration on the Dynamics of *V. microstoma* Polymorphic Stages

At room temperature (25 ± 2°C) in aqueous condition, only the live sessile stalked trophont stage of *V. microstoma* was observed (Figure 1(a)). However, under dehydrated conditions with no water at room temperature (25 ± 2°C), only the cyst stage of *V. microstoma* was observed (Figure 1(f)) after 24 h of exposure. The cyst stage was round in its shape, without a contractile stalk, and had a clearly visible membrane around the cyst. In contrast, at 11°C under aqueous conditions, five different morphological stages of *V. microstoma* were observed: live sessile stalked trophont stage (Figure 1(a)), consisting of a detached cup-like body with a short, mobile stalk (Figure 1(b)); detached cup-like body without a mobile stalk (Figure 1(c)); an intermediate stage to telotroch stage (Figure 1(d)); telotroch stage, which is highly mobile (Figure 1(e)); the immobile cyst stage (Figure 1(f)). The detached cup-like bodies with a short, mobile stalk or without a stalk were freely-swimming stages. The telotroch stage was elongated and had a long cylinder-shaped body, without a contractile stalk. They had a posterior girdle of cilia and was a freely-swimming stage. Under these conditions, the telotroch stage was predominant. Exposure to 11°C and dehydration resulted in the presence of only the cyst stage of *V. microstoma*, which is immobile (Figure 1(f)). The reduction of temperature to 6°C, with or without water, caused only the immobile cyst stages of *V. microstoma* to be observed (Figure 1(f)).

### 3.4. Viability of the Cyst Stage of *V. microstoma* under Prolonged Dry Conditions

After 24 h of exposure of *V. microstoma* in petri dishes to continuous dry conditions, only the immobile cyst stages were observed (Figure 1(f)). However, they were transformed to its trophont stage after addition of water and observing for 24 h. The transformation of the cyst stage into the trophont stage was observed up to 21 d. This indicates that under waterless dry conditions, the cyst stage of *V. microstoma* can survive up to 21 days under laboratory conditions.

## 4. Discussion

A study conducted by Patil et al. [11] revealed that the inhibition of larval growth, development, and adult emergence of *An. stephensi* larvae due to infection of *Vorticella* sp. Far back in 1950, Mick [9] reported the lethal effect of the ciliate, *V. microstoma* on *An. quadrimaculatus*, while the present study reveals the lethal effect of *V. microstoma* on *Cx. tritaeniorhynchus*, *Cx. gelidus*, *Cx. quinquefasciatus*, and *An. subpictus* mosquito larvae. The reason for the death of mosquito larvae due to attachment of *Vorticella* is still not well understood. Mick [9] stated that the larval death may be apparent due to the inability of the infected larvae to remain on the water surface, thereby interfering with respiration and drowning. However, Patil et al. [11] presumed that the organism secretes some biochemical substances to fix itself on the substrate, and those substances may damage some surface sensory system or cause pore formation in the larval body. It is also possible that the metabolic and secretory products of *Vorticella* are toxic to the mosquito larvae, polluting its natural environment [11]; hence, the use of *Vorticella* has been explored as a biocontrol strategy for mosquitoes. The present study reveals the drowning of moribund mosquito larvae in water. It is also possible that the metabolic and secretory products of *Vorticella* species are toxic to mosquito larvae [11]. Besides mosquito larvae, *Vorticella* species have been found attached to the integument of nematodes, tardigrades, and chironomids as well and the nematodes within fresh extracts from soil samples. *Vorticella* attached to the cuticle of nematodes was found to be moving initially but gradually became sluggish and finally died 18-24 hours after isolation [19].

In this study, *V. microstoma* trophonts did not usually attach to the siphon region of live mosquito larvae, most probably due to the hardness of the cuticle. However, they attached to the siphon region once the infected mosquito larvae are dead possibly owing to the reduction of the thickness of the siphon cuticular layer due to autolysis. *Ae. albopictus* and *Ae. aegypti* did not show any infestation or mortality due to *V. microstoma* in the present study. The reason underlying this is not clearly evident. Patil et al. [11] quoted from an unrecorded reference that *Vorticella* infection was found only in *Anophele*s spp., and infection or mortality was not observed in *Ae. aegypti*. However, their study showed that *Vorticella* sp. prefers *Anophele*s and suggested that attachment that it prefers other mosquito species such as *Ae. aegypti* as a second preference.

It has long been recognized that different species of *Vorticella* often have a predilection for different ecological conditions [12]. *V. microstoma* species sometimes stay in clusters or groups considered as pseudocolonies, but they are not true colonies because each cell has its own individual stalk. This allows it to detach from the cluster at any time, usually by reverting to a telotroch stage when environmental conditions are unfavorable *V. microstoma* also swim freely if they have to detach themselves from the substrate due to unfavorable condition. Thus, the sessile form can transform into a telotroch stage and becomes free-swimming in search of a congenial environment. The present study also reveals the encystation of *V. microstoma* under desiccation and excystation again when reflooding the cysts formed. The original ciliate sample was collected from a paddy field during the present study, As there are two major paddy transplantation seasons in Sri Lanka, coupled with two monsoon rain types; after the paddy is harvested, vector breeding habitats got limited with the restricted distribution of parasitic or pathogenic ciliates in host mosquito larvae. Thus, survival of the parasitic agent should undergo under dry conditions until the next season of paddy transplantation coupled with monsoon rains under high vector density situation returns. The encystation of *V. microstoma* seems a possible way for the time-lap of the dry season until the next paddy plantation period occurs with rain. After excystation, as *V. microstoma* has a high reproductive potential, the number of trophonts could be increased easily when the optimum environmental conditions reoccurred. Cysts and the processes of encystation and excystation have been described for *V. microstoma* [20].

Likewise, prolonged exposure to induced unfavorable conditions, such as temperature reduction, forced *V. microstoma* to transform through the telotroch stage to the cyst stage in this study. The cyst stage in the aqueous situation could transform to the trophont stage through the telotroch stage, and the cysts in prolonged dry conditions “without water” were able to survive up to 21 days.

When the food is exhausted, they got excysted, and the addition of bacteria cause also to excyst or to increase the size, and found with a higher multiplication rate under the presence of bacteria [21]. When starved, the ciliate is thinner, thus gets more elongated. When a trophont is well-fed with bacteria, trophont becomes swollen and striations in the body get no longer visible. The encysted *V. microstoma* gets commences with the formation of the contractile vacuole which pulsates, and the ciliate excysted from the cyst membrane through a cyst-pore, forced open by hydrostatic pressure due to the activity of the contractile vacuole. It took nearly an hour from signs of a contractile vacuole to the escape of the ciliate from the cyst membrane. Then, the escaped individual turns in to a telotroch, and then to sessile trophont [21].

Epibiont ciliates make up a significant part of the biomass in aquatic ecosystems and may cause perceptible alterations in the population dynamics of their hosts. A study carried out by Cabral et al. [21] found that the *Chironomus* genus, of which 16.95% were colonized by *Rhabdostyla* aff. *chironomi*, colonizing the chironomids' ventral tubules. The high number of chironomid larvae, high host- and site-specificity, low infestation intensity, and absence of apparent structural damage to hosts evidence an intimate relationship between epibiont and basibiont. But compared to that, epibiont interaction of *V. microstoma* with mosquito larvae, a host specificity was found, and the rate of infection with the epibiont was dependent on that [22]. The ciliate, *Chilodonella uncinata* was found to cause 25–100% of mortalities in larval stages of the JE vectors in North India and widely distributed in typical JE vector-breeding habitats [23]. Further, anopheline larvae found less susceptible to *Chilodonella* infection than culicine larvae revealing that this ciliate also has a different degree for pathogenism and host selection and host specificity over different species of mosquito larvae [23]. Therefore, host specificity seems to be an important factor for the degree of ciliate infections.

In the field of applied ecology, there have been many attempts to achieve the biological control of pathogens or vectors by introducing new effective natural enemies to their natural habitats [24]. The efficient selection of effective natural enemies has become increasingly important for the success of biological control programs. The selection of biological control agents should be based on their potential for unintended impacts, self-replicating capacity, climatic compatibility, and their capability to maintain very close interactions with target populations [25]. Also, the biological controlling agent's adaptability to the introduced environment and overall interaction with indigenous organisms need to be considered prior to the introduction [26]. Until recently, the ecological role of environmental managers has been more concentrated on preventing damage from pollution rather than proposing sustainable solutions to different global and local problems faced by human societies. One of the multiple possibilities of applying ecological theories for human welfare is the use of our knowledge about the effects and mechanisms of predation, parasitism, and competition within various kinds of permanent and temporary aquatic habitats. By manipulating particular trophic levels, desired changes can be achieved in a system [27].

However, the application of *Vorticella* as a biocontrol agent should be further investigated because *Vorticella* sp. are ectocommensals that are prevalent in freshwater shrimps, attaching independently to their rostrum, gills, and appendages, acting as a parasite. Some species of *Vorticella* have also been reported among cultured tilapia in several farms in Saudi Arabia [28].

## 5. Conclusions

Present findings would be considered as a first step and basic information found for a new-avenue to work on mosquito larval controlling in an environmentally agreeable manner. *V. microstoma* studied in this work readily attach to *Culex* species mosquito larvae causing death in *Cx. tritaeniorhynchus* (100%) and *Cx. gelidus* (70%) in 48 hours. However, this organism did not attach to *Aedes* species mosquito larvae and result in the death of *Ae. albopictus* and *Ae. aegypti* under the same experimental condition. *V. microstoma* used in this study acted as a good biocontrol agent against *Culex* larvae. Induced unfavorable conditions caused for the different morphological forms of *V. microstoma* and their encystation and excystation may appear as a better way for a time-lap under stressed changes in the environment.

## Figures and Tables

**Table 1:** Multiple comparison between mortality percentages of mosquito species (IBM SPSS Statistics 22 software).

Replicate (I)	Replicate (J)	Mean difference (I-J)	Std. error	Significant level	95% confidence interval	
					Lower bound	Upper bound
*Cx. tritaeniorhynchus*	*Tripteroides* spp.	53.33^∗^	14.40	0.01	18.09	88.57
	*Ae. albopictus*	100.00^∗^	14.40	0.00	64.76	135.24
*Tripteroides* spp.	*Cu. tritaeniorhynchus*	-53.33^∗^	14.40	0.01	-88.57	-18.10
	*Ae. albopictus*	46.67^∗^	14.40	0.02	11.43	81.91
*Ae. albopictus*	*Cx. tritaeniorhynchus*	-100.00^∗^	14.40	0.00	-135.24	-64.76
	*Tripteroides* spp.	-46.67^∗^	14.40	0.02	-81.91	-11.43

^∗^The differences between mean values are significant at the 0.05 level.

**Table 2:** Multiple comparison between mortality percentage of different instars of *Cx. gelidus* larvae (IBM SPSS Statistics 22 software).

Replicate (I)	Replicate (J)	Mean difference (I-J)	Std. error	Significant level	95% confidence interval	
					Lower bound	Upper bound
1^st^ instar	2^nd^ instar	-13.33	16.73	0.46	-54.27	27.61
	3^rd^ instar	15.56	16.73	0.39	-25.38	56.50
2^nd^ instar	1^st^ instar	13.33	16.73	0.46	-27.61	54.27
	3^rd^ instar	28.89	16.73	0.14	-12.05	69.83
3^rd^ instar	1^st^ instar	-15.56	16.73	0.39	-56.50	25.38
	2^nd^ instar	-28.89	16.73	0.14	-69.83	12.05

## Data Availability

The datasets supporting the conclusions of this article are included in the article. Data will not be shared in any of the sources.
